# The Role of Adenosine Receptor Agonists in Regulation of Hematopoiesis

**DOI:** 10.3390/molecules16010675

**Published:** 2011-01-17

**Authors:** Michal Hofer, Milan Pospisil, Lenka Weiterova, Zuzana Hoferova

**Affiliations:** Working Group of Experimental Hematology, Institute of Biophysics, v.v.i., Academy of Sciences of the Czech Republic, Královopolská 135, CZ-61265 Brno, Czech Republic

**Keywords:** adenosine receptors, hematopoiesis, myelosuppression

## Abstract

The review summarizes data evaluating the role of adenosine receptor signaling in murine hematopoietic functions. The studies carried out utilized either non-selective activation of adenosine receptors induced by elevation of extracellular adenosine or by administration of synthetic adenosine analogs having various proportions of selectivity for a particular receptor. Numerous studies have described stimulatory effects of non-selective activation of adenosine receptors, manifested as enhancement of proliferation of cells at various levels of the hematopoietic hierarchy. Subsequent experimental approaches, considering the hematopoiesis-modulating action of adenosine receptor agonists with a high level of selectivity to individual adenosine receptor subtypes, have revealed differential effects of various adenosine analogs. Whereas selective activation of A_1_ receptors has resulted in suppression of proliferation of hematopoietic progenitor and precursor cells, that of A_3_ receptors has led to stimulated cell proliferation in these cell compartments. Thus, A_1_ and A_3 _receptors have been found to play a homeostatic role in suppressed and regenerating hematopoiesis. Selective activation of adenosine A_3_ receptors has been found to act curatively under conditions of drug- and radiation-induced myelosuppression. The findings in these and further research areas will be summarized and mechanisms of hematopoiesis-modulating action of adenosine receptor agonists will be discussed.

## 1. Introductory Remarks to the Problem of Adenosine Receptor Signaling

In 1929, Drury and Szent-Györgyi [1] described striking cardiovascular effects of adenosine injected into mammals. Since that time ample evidence has accumulated suggesting the regulatory action of adenosine in many functions and organ systems of the mammalian organism. Adenosine, a naturally occurring nucleoside, is formed in the body by degradation of adenine nucleotides. It can be formed intracellularly as well as extracellularly but acts in a regulatory sense only on extracellular receptors. Such an action of adenosine seems to be phylogenetically ancient and represents a universal intracellular communication system in plants and animals [2]. As stated by Linden [3], “adenosine is a primordial signaling molecule that has evolved to modulate physiological responses in all mammalian tissues”. In general, the action of adenosine can be designated as protective and homeostatic. This central paradigm of adenosine research is based on the hypothesis of Berne [4] that adenosine is the metabolite that increases blood flow during hypoxia, Newby’s [5] concept denoting adenosine as a retaliatory metabolite, and Brun’s idea [6] that adenosine controls energy supply/demand balance by reducing energy demand and increasing energy supply. An important turning-point in adenosine research has been the discovery and description of adenosine receptors that are integral membrane molecules for binding extracellular adenosine agonists and can initiate the transmembrane signal to activate the second messenger system. Up to date four subtypes of adenosine receptors have been described, namely A_1_, A_2a_, A_2b_, and A_3_. Activation of these receptors can be achieved either non-selectively by the endogenous agonist adenosine, or selectively by use of various adenosine analogs exhibiting different degrees of receptor specificity. The potency of adenosine receptor agonists is dependent not only on the type of the receptor activated but also on the expression of the individual receptor subtypes which may be supposed to possess the ability to change in dependence on the functional state of the cell population investigated. In addition, it has to be taken into account that more adenosine receptor subtypes can be present on the same cell [7,8,9,10,11,12].

The aim of this review is to summarize the published data related to the effects of adenosine receptor signaling on various parameters of murine hematopoiesis, including quantitative indices of various compartments of the bone marrow, as well as the proliferation status and regeneration potential of hematopoietic cell populations investigated under conditions of the steady state, myelosuppression, and regeneration. Attention has been also given to the problem of interactions of control mechanisms based on adenosine receptor signaling and action of hematopoietic cytokines. It has to be noted that the range of problems reviewed here does not include those dealing with adenosine receptor influence on functions of mature peripheral blood cells and on immune and inflammatory functions [13,14].

## 2. Hematological Effects of Non-Selective Activation of Adenosine Receptors

Adenosine can interact with a cell either by a mechanism not mediated by receptors (by a direct membrane uptake) or by a receptor-mediated mechanism. Adenosine is a natural endogenous adenosine receptor agonist and pharmacologically induced elevation of extracellular adenosine can lead to an enhancement of non-selective receptor-mediated adenosine action. Dipyridamole, a drug inhibiting the cellular uptake of adenosine [15,16], has been used in studies on a number of cell types [17,18,19,20] for obtaining higher extracellular adenosine levels, for discrimination between receptor-mediated and non-receptor-mediated mechanisms of adenosine actions, and/or for potentiation of the receptor-mediated mode of adenosine functioning. Less attention has been paid to the effects of elevated extracellular adenosine levels on various populations of the hierarchically organized hematopoietic cell system, which is under the control of a complex regulatory network. In the published studies on mice, elevated extracellular adenosine levels and thereby evoked enhancement of non-selective activation of adenosine membrane receptors have been achieved by combined administration of dipyridamole and adenosine monophosphate. Adenosine monophosphate (AMP), which is rapidly metabolized extracellularly to adenosine by cell-surface ectonucleotidase [21], has served as an adenosine prodrug having a higher solubility in water as compared with adenosine. Therefore, AMP is more convenient for *in vivo* administration because its administration makes it possible to avoid loading the mice with a large volume of fluid. It should be noted that doses of dipyridamole and AMP used in the experiments described in this review exhibit significant pharmacological effects at low toxicity [22]. The efficiency of the combined action of the drugs compared to their action when given alone has been verified [23].

First suggestions concerning the role of extracellular adenosine in modulation of hematopoietic functions under *in vivo* conditions when using the combined administration of dipyridamole and AMP for increasing its levels were brought by radiobiological experiments aimed at the assessment of radiation-induced cellular damage. The combination of dipyridamole and AMP administered several minutes before whole-body gamma-irradiation of mice decreased cellular damage as revealed by the thymidine level in plasma and the amount of free polynucleotides in the thymus and spleen [24]. Similar effects of the drug combination were found when administering the drugs immediately after irradiation [25]. In addition, it has been ascertained that pre-irradiation administration of the drugs decreases the formation of micronucleated polychromatic erythrocytes, an indicator of the genetic radiation damage [26]. Mechanisms of the effects of adenosine receptor participation on these early indices of cell radiosensitivity are not clear. However, it seems that the short-termed reduction of the blood pressure due to bradycardia and vasodilation, which are known effects of the receptor action of extracellular adenosine [27] leading to transient systemic hypoxia, can be responsible for this effect; hypoxia-induced radioprotection is a classical radiobiological phenomenon. Another alternative can be the enhancement of the mechanisms of intracellular repair: it was reported that the addition of the drugs elevating extracellular adenosine to irradiated thymocytes enhanced the rejoining processes of DNA strand breaks [25].

Further experiments investigated the manifestations of the postirradiation bone marrow syndrome, *i.e.* suppression of hematopoiesis in sublethally irradiated mice, when administering the drugs in the protective regimen, *i.e.* before irradiation. Survival of animals irradiated with absolute lethal doses under this drug treatment regimen has been evaluated, as well. These experiments confirmed the protective action of dipyridamole and AMP combination under conditions of single [23,27,28,29] and also fractionated irradiation [30,31]. These effects are most probably due to the modulation of regulatory functions, *i.e.* to the stimulatory action of adenosine receptor signaling on hematopoiesis. This has been suggested on the basis of experiments utilizing administration of noradrenaline which reduces cardiovascular and thus hypoxia-inducing effects of the combination of dipyridamole and AMP [28]. Furthermore, results of experiments prolonging the time interval between the administration for the drug combination and irradiation in order to avoid the operation of hypoxia during the radiation exposure have also supported the above hypothesis [31]. In both these situations protective action of adenosine receptor signaling occurring in hematopoiesis and postirradiation survival of animals was preserved.

In several studies attention was focused at the action of extracellular adenosine in normal and myelosuppressed mice. It has been found that administration of dipyridamole and AMP to normal mice induces pleiotropic amplification effects in cell compartments of the bone marrow and spleen [22,23], enhances cycling of committed hematopoietic progenitor cells [32] and mobilizes these cells into peripheral blood [33]. The possibility that the elevation of extracellular adenosine activates mechanisms of the positive control of hematopoiesis has been also supported by findings demonstrating curative effects of the drugs administered repeatedly under conditions of myelosuppression induced in mice by irradiation [34], cytostatic drugs [35,36] and combination of both these myelosuppressive actions [37]. Enhancement of the regeneration of hematopoiesis by the combination of drugs elevating extracellular adenosine has been found in all these experiments.

Previously it has been shown that adenosine signaling can act in concert with conventional growth factors, cytokines and other growth regulatory molecules to modulate cell functions in various non-hematopoietic cell systems [2]. Such effects have been also observed in studies investigating the effects of combined administration of drugs elevating extracellular adenosine with granulocyte colony-stimulating factor (G-CSF) on hematopoiesis. Mutual potentiation of granulopoiesis-stimulating effects of these agents has been observed in normal mice [22,33] and in mice treated under conditions of myelosuppression induced by radiation [34,38], cytostatic drugs [36], or a combination of both these myelosuppressive actions [37]. These results might have a clinical impact, especially in chemotherapy and radiotherapy.

## 3. Hematological Effects of Selective Activation of Adenosine Receptors

To obtain more detailed information about mechanisms of extracellular, receptor-mediated adenosine action on hematopoiesis and to uncover new therapeutic possibilities of treatment of hematopoietic suppression by activating adenosine receptors, further experimental steps should be carried out taking into account the known adenosine receptor diversity. Discrimination between adenosine receptors was made possible when more or less selective adenosine receptor agonists became available. These agents, adenosine analogs, do not undergo facilitated uptake nor represent a substrate for cellular metabolism and are relatively resistant to extracellular adenosine-metabolizing enzymes [39]. Their serum half-lives are in the order of minutes [40,41]; however, as it will be shown below, these rather short half-lives do not prevent adenosine analogs from being functional. In our recent experiments investigating murine hematopoiesis, three synthetic adenosine analogs, selective for A_1_, A_2a_, and A_3_ adenosine receptors, *i.e.* *N*^6^-cyclopentyladenosine (CPA), 2-*p*-(2-carboxyethyl)-phenethylamino-5’-*N*-ethylcarboxamidoadenosine (CGS 21680), and *N*^6^-(3-iodobenzyl)adenosine-5’-*N*-methyluronamide (IB-MECA), respectively, have been compared in terms of their ability to modulate cell cycling in hematopoietic progenitor bone marrow cells as inferred from the action of the cell cycle-specific drug 5-fluorouracil (5-FU) administered to mice *in vivo* [42]. The most interesting findings of these experiments have been the opposite effects of the selective agonists for A_1_ and A_3_ receptors, *i.e.* of CPA and IB-MECA, manifesting themselves as inhibitory or stimulatory actions on cell cycling in the compartments of the bone marrow progenitor cells. In terms of their signaling pathways, both these receptors inhibit adenylyl cyclase [43], but it has been reported that they differ in their coupling to phospholipases C and D [44]. Further studies comparing the effects of these two selective agonists on states of myelosuppression and regeneration induced by 5-FU have confirmed marked stimulatory action of IB-MECA administered in the phase of cell depletion and its ineffectiveness when given in the phase of cell recovery [45]. However, the agonist of adenosine A_1_ receptors, CPA, has been found to be effective as inhibitor of cell cycling when administered in the phase of cell regeneration [46]. These effects suggest the homeostatic role of adenosine receptor signaling when influencing hematopoiesis. The effects observed might be logically explainable as evidence of the changing expression of different adenosine receptors during various functional states of the investigated cell systems [47,48]. Our findings on the hematopoiesis-stimulating effect of pharmacological activation of adenosine A_3_ receptors confirm earlier observations of Merimsky *et al.* [49] on myelostimulatory effects of IB-MECA in cyclophosphamide-treated mice.

## 4. Adenosine Receptors—Participation in Various Signaling Pathways

Until recently, only findings on the expression of adenosine receptor mRNAs in isolated mature human peripheral blood cells have been reported: all four subtypes of adenosine receptors have been described in human neutrophils [50] and monocytes [51], A_2a_, A_2b_, and A_3_ receptors have been found in human lymphocytes [52,53,54]. As late as in 2010, our laboratory has reported that mRNAs of all four subtypes of adenosine receptors are expressed, though in different relative amounts, in differentiating and proliferating cells of various hematopoietic cell compartments, namely in mouse bone marrow granulopoietic/monocytopoietic, erythropoietic and B-lymphopoietic cells, as well as T-lymphopoietic cells in thymus [55]. However, these data represent only a limited information related to the resting and balanced state of the hematopoietic system. As shown in the preceding paragraph, expression of receptors deduced from different actions of various agonists is supposed to be variable and in accordance with the requirements of the system to maintain homeostasis. Furthermore, it is important to note that the effects of the actions of adenosine receptor agonists on hematopoietic cells can be influenced additively by activation of adenosine receptors in the cells of the supportive microenvironment in the hematopoietic tissues. Macrophages are known to represent an important constituent of the hematopoietic microenviroment and to produce hematopoiesis-stimulating factors like e.g. interleukin-6 and G-CSF [56,57]. Recent studies on mouse macrophages have shown that whereas the mRNA expression of adenosine A_1_ receptors in both normal and lipopolysaccharide-activated cells is very low and unquantifiable, the other three receptor subtypes are expressed at various but always quantifiable levels [58,59]. Other marrow-derived cells, including fibrocytes and endothelial precursor cells are also influenced by modulating the activity of A_2a_ adenosine receptors [60,61,62]. In addition to these data it is appropriate to mention results of experiments demonstrating that the serum of mice, which received drugs increasing extracellular adenosine, exhibited enhanced ability to support proliferation of granulocyte-macrophage colony-forming cells *in vitro* and to elevate serum levels of interleukin-6 [63]. Worth mentioning are also experiments of Bar-Yehuda *et al.* [64] who have described a stimulatory effect of the agonist of A_3_ receptors, IB-MECA, on production of G-CSF in mice receiving this drug per os for several days. Interesting findings have been reported on the positive interaction of the agonist of adenosine A_3_ receptors with the growth factors interleukin-3 and stem cell factor that influenced the growth of hematopoietic progenitor cells for granulocytes and macrophages in suspension of normal murine bone marrow cells *in vitro* [65]. These data indicate the possibility of cooperation and/or coalescence of the two different signaling pathways into a common pathway even under *in vivo* conditions. Indeed, combined treatment of the γ-irradiated mice with the agonist of adenosine A_3_ receptors IB-MECA and G-CSF induced the highest recovery of hematopoiesis as compared with the effects induced by the drugs administered alone [66]. The above-mentioned topics are quite open and deserve further research.

## 5. Possibilities of Practical Utilization of Adenosine Receptor Agonists in Clinical Hematology

The results on hematopoiesis-stimulating effects of IB-MECA, the selective A_3_ adenosine receptor agonist, suggest the possibility to use this drug in clinical practice as a part of the spectrum of pharmacological approaches aimed at treating myelosuppression of various etiology. This view is supported by the fact that IB-MECA is at present available as a good manufacturing practice (GMP) grade for peroral use [67]. Currently investigated directions of possible therapeutic applications include not only myelostimulatory effects of IB-MECA but also antitumor, cardioprotective, neuroprotective, and antiinflammatory actions of the drug [68], the latter ones having passed already through a phase II clinical trial [69]. Therefore, the way to the use pharmacological activation of adenosine A_3 _receptor may prove to be a promising approach in clinical hematology.
